# CRISPR-Cas9-Based Toolkit for *Clostridium botulinum* Group II Spore and Sporulation Research

**DOI:** 10.3389/fmicb.2021.617269

**Published:** 2021-01-27

**Authors:** Anna Mertaoja, Maria B. Nowakowska, Gerald Mascher, Viivi Heljanko, Daphne Groothuis, Nigel P. Minton, Miia Lindström

**Affiliations:** ^1^Department of Food Hygiene and Environmental Health, Faculty of Veterinary Medicine, University of Helsinki, Helsinki, Finland; ^2^Clostridia Research Group, BBSRC/EPSRC Synthetic Biology Research Centre (SBRC), Biodiscovery Institute, School of Life Sciences, University of Nottingham, Nottingham, United Kingdom

**Keywords:** *Clostridium botulinum* Group II, CRISPR-Cas9, sporulation medium, *spo0A*, spore

## Abstract

The spores of *Clostridium botulinum* Group II strains pose a significant threat to the safety of modern packaged foods due to the risk of their survival in pasteurization and their ability to germinate into neurotoxigenic cultures at refrigeration temperatures. Moreover, spores are the infectious agents in wound botulism, infant botulism, and intestinal toxemia in adults. The identification of factors that contribute to spore formation is, therefore, essential to the development of strategies to control related health risks. Accordingly, development of a straightforward and versatile gene manipulation tool and an efficient sporulation-promoting medium is pivotal. Our strategy was to employ CRISPR-Cas9 and homology-directed repair (HDR) to replace targeted genes with mutant alleles incorporating a unique 24-nt “bookmark” sequence that could act as a single guide RNA (sgRNA) target for Cas9. Following the generation of the sporulation mutant, the presence of the bookmark allowed rapid generation of a complemented strain, in which the mutant allele was replaced with a functional copy of the deleted gene using CRISPR-Cas9 and the requisite sgRNA. Then, we selected the most appropriate medium for sporulation studies in *C. botulinum* Group II strains by measuring the efficiency of spore formation in seven different media. The most effective medium was exploited to confirm the involvement of a candidate gene in the sporulation process. Using the devised sporulation medium, subsequent comparisons of the sporulation efficiency of the wild type (WT), mutant and “bookmark”-complemented strain allowed the assignment of any defective sporulation phenotype to the mutation made. As a strain generated by complementation with the WT gene in the original locus would be indistinguishable from the parental strain, the gene utilized in complementation studies was altered to contain a unique “watermark” through the introduction of silent nucleotide changes. The mutagenesis system and the devised sporulation medium provide a solid basis for gaining a deeper understanding of spore formation in *C. botulinum*, a prerequisite for the development of novel strategies for spore control and related food safety and public health risk management.

## Introduction

*Clostridium botulinum* is a Gram-positive, strictly anaerobic, spore-forming bacterium. *C. botulinum* strains are traditionally classified into four genetically and physiologically diverse groups designated I–IV (Hatheway, 1993). The critical characteristic of all four groups is their ability to produce extremely potent neurotoxin which is the causative agent of botulism, a rare but deadly disease affecting humans and animals (Sobel, 2005). *C. botulinum* strains of Group I and Group II are usually associated with human botulism, which classically manifests as a food poisoning. This form of botulism results from consumption of foods where *C. botulinum* spores germinated and outgrew into a neurotoxigenic culture. Their toxinogenic potential at refrigeration temperatures makes *C. botulinum* Group II strains a particular food safety concern (Lindström et al., 2006). Moreover, *C. botulinum* spores can colonize wounds or the digestive tract of infants and susceptible adults with compromised gut microbiota, causing the toxicoinfectious forms of wound and intestinal botulism, respectively (Harris, 2015).

Any interventions for controlling the risks posed by *C. botulinum* spores rely on the fundamental understanding of the spore composition and resistance properties, as well as the processes of spore formation, spore germination, and neurotoxin production. High-quality mechanistic research is reliant on precise deletions, genome manipulations, and efficient screening assays for the targeted phenotypes. Some progress has been made in recent times as a consequence of the development of appropriate genome editing tools (Heap et al., 2007; Ng et al., 2013; Minton et al., 2016; Cañadas et al., 2019; Ingle et al., 2019; Kuehne et al., 2019). These include the extensively utilized ClosTron together with various allelic exchange methodologies which have been exploited to successfully construct insertion and deletion mutants of sporulation and toxin-related genes in *C. botulinum* (Dahlsten et al., 2013; Kirk et al., 2014; Mascher et al., 2014, 2017; Zhang et al., 2014; Clauwers et al., 2016, 2017). The ClosTron, however, like any insertional mutagen is compromised by the fact that mutants may have altered phenotypes due to polar effects on genes located downstream of the intron insertion (Heap et al., 2007). Although polar effects may be overcome using allelic exchange to make in-frame, markerless deletion mutants (reviewed in Minton et al., 2016), the strategy utilized so far in *C. botulinum* Group II strains has resulted in the insertion of an antibiotic resistance gene (Clauwers et al., 2016, 2017). To make markerless deletions, counterselection markers are necessary, such as *pyrE* and *codA* (Minton et al., 2016), but their deployment requires the use of minimal media. Finding a minimal medium that is applicable to metabolically diverse *C. botulinum* strains is, however, challenging (Whitmer and Johnson, 1988).

Recently, the ease with which allelic exchange may be deployed in clostridia, including *C. botulinum* Group I strains, has been considerably improved through the exploitation of clustered regularly interspaced short palindromic repeats-CRISPR-associated protein 9 (CRISPR-Cas9; Cañadas et al., 2019; Ingle et al., 2019; McAllister and Sorg, 2019). Using such an approach, the allelic-exchange driven gene deletion makes the resulting mutant cells immune to Cas9-cleavage. This enables direct selection of mutant colonies, as opposed to having to be passaged onto specialized media where counterselection markers are employed. Consequently, the reduced number of steps leading to the mutant isolation decreases the likelihood of accumulating undesired ancillary mutations [single nucleotide polymorphisms (SNPs) and/or insertion/deletions (InDels)] that may affect phenotype. Thus, CRISPR-Cas9-based methods offer distinct advantages for generating mutants in *C. botulinum* Group II strains.

Another critical component in the fundamental spore research is a medium that efficiently promotes sporulation and ensures sufficient spore yields for downstream applications. While *Bacillus* spp. sporulation is mainly induced by starvation (Grossman and Losick, 1988), the factors triggering clostridial sporulation appear more complex (Perkins and Tsuji, 1962; Tsuji and Perkins, 1962; Roberts, 1965, 1967; Strasdine and Melville, 1968; Hawirko et al., 1979). Furthermore, the four different *C. botulinum* groups have distinct physiology and metabolism and therefore they respond differently to environmental cues (Woods and Jones, 1987) and assumed triggers of sporulation (Roberts, 1967; Solomon and Kautter, 1979). The routine growth media used for *C. botulinum* Group II strains yield insufficient numbers of spores for downstream applications [e.g., tryptone-peptone-glucose-yeast extract broth (TPGY) 10^5^ spores/ml (Mascher et al., 2017)]. Several studies have shown evidence that biphasic media consisting of a solid agar phase and a liquid water phase can support efficient sporulation of Group II strains. However, the solid phase is prone to crumbling and thus makes sampling for many downstream applications difficult (Bruch et al., 1968; Peck et al., 1992). In order to facilitate spore biology research in *C. botulinum* Group II, a medium triggering efficient sporulation and enabling easy sampling is urgently needed.

Here we took the opportunity to exemplify CRISPR-Cas9 “bookmark” technology, a recently proposed concept (Seys et al., 2020) for gold standard complementation studies, which exploits CRISPR-Cas9 to replace the mutant allele *in situ* with a wild type (WT) allele. Moreover, we established a novel medium for the characterization of spore formation in *C. botulinum* Group II strains and used it to determine the suitability of a previously described CRISPR-Cas9 system (Ingle et al., 2019) for spore mutant generation in these strains. To exemplify these systems, we targeted the *spo0A* gene of *C. botulinum* Group II strain Beluga as its inactivation has been shown to result in an asporogenous phenotype (Al-Hinai et al., 2015; Mascher et al., 2017). The exemplified procedures form a solid basis for future spore-related studies in *C. botulinum* Group II strains.

## Materials and Methods

### Experimental Design

The study consisted of four parts: (1) sporulation mutants of *C. botulinum* Group II type E strain Beluga were constructed using a novel CRISPR-Cas9 bookmark approach (Seys et al., 2020). (2) Growth and sporulation of WT Beluga was tested in seven different biphasic media. The spore and viable cell counts were enumerated directly after inoculating the medium, and subsequently at 1 and 6days after inoculation. (3) Growth and sporulation of three different Group II strains (Beluga, Eklund 17B, FT10F) were tested in cooked meat medium-TPGY (CMM-TPGY), which was the medium selected as best meeting the study criteria in (2), and in two control media: TPGY and Duncan-Strong (DS), the latter supporting the sporulation of *Clostridium perfringens* (Duncan and Strong, 1968). Spore and viable cell counts were enumerated directly after inoculating the medium, and subsequently at 1day, 1week, and 2weeks after inoculation. Phase-contrast microscopy of the cultures was performed 2weeks after inoculation. (4) The mutant strains constructed in (1) were characterized and validated in CMM-TPGY. Spore and viable cell count assays and phase-contrast microscopy were performed as in (3).

### Strains, Media, and Growth Conditions

All the *C. botulinum* and *Escherichia coli* strains used are listed in Supplementary Table S1. *C. botulinum* cultures were routinely grown at 30°C in an anaerobic workstation (MG1000 anaerobic workstation; Don Whitley Scientific Ltd., Shipley, United Kingdom) with an atmosphere of 85% N_2_, 10% CO_2_ and 5% H_2_. Spore or glycerol stocks of *C. botulinum* strains were revived in TPGY broth composed of 5% (w/v) tryptone, 0.5% peptone, 2% yeast extract (Difco, BD Diagnostic Systems, Sparks, MD), 0.4% glucose (VWR Chemicals, Leuven, Belgium), and 0.1% sodium thioglycolate (Merck, Darmstadt, Germany) by inoculating 5ml of the medium with either 5μl of spore stock or 50μl of glycerol stock and incubating until growth reached stationary phase. An overnight culture was prepared by inoculating 5ml of TPGY with 50μl of revived culture. All the media used for *C. botulinum* growth experiments were deoxygenated prior to use, either by boiling for 20min or by exposure to anaerobic conditions for at least 24h. All tests were run in triplicate.

In part 1 of the study, transformed strains were grown on TPGY plates containing 1.5% (w/v) bacteriological agar supplemented with 250μgml^−1^ cycloserine (Merck) and 15μgml^−1^ thiamphenicol (Merck). *E. coli* strains used for cloning and conjugation procedures were grown in Luria-Bertani (LB; Invitrogen, Carlsbad, CA) broth or on LB agar plates incubated aerobically at 37°C. For molecular cloning, LB was supplemented with 25μgml^−1^ chloramphenicol (Merck) and for conjugation with 25μgml^−1^ chloramphenicol and 30μgml^−1^ kanamycin (Merck).

In part 2 of the study, *C. botulinum* strain Beluga was grown in seven different biphasic media: (i) egg-yolk-agar (EYA)-H_2_O consisting of a solid EYA phase [2% peptone, 0.5% yeast extract, 0.5% tryptone, 0.5% NaCl, 10% (v/v) egg yolk emulsion (Oxoid Microbiology Products, Basingstoke, United Kingdom), and 1.5% agar] and a liquid phase of sterile water; (ii) TPGY-H_2_O consisting of a solid TPGY agar phase (TPGY broth with 1.5% bacteriological agar (Amresco, Ohio, United States) and a liquid phase of sterile water; (iii) Reinforced Clostridial Medium (RCM)-H_2_O consisting of a solid RCM phase [Reinforced Clostridial Medium (Lab M limited, Lancashire, United Kingdom) prepared according to manufacturer’s instructions, and 1.5% bacteriological agar] and a liquid phase of sterile water; (iv) CMM)-H_2_O consisting of a solid phase of 10% (w/v) cooked meat medium (Oxoid) with 0.1% glucose and 1.5% agar, and sterile water; (v) CMM-TPGY consisting of a solid CMM phase as in (iv) and a liquid phase of TPGY broth; (vi) agar-TPGY consisting of a solid phase of 1.5% agar and a liquid phase of TPGY broth, and (vii) agar-H_2_O consisting of a solid phase of 1.5% agar and a liquid phase of sterile water. All biphasic media used in part 2 had a 150-ml solid phase and a 20-ml liquid phase. The liquid phase was inoculated with 5ml of overnight culture in TPGY to a total volume of 25ml.

In part 3 of the study, Group II strains Beluga, Eklund 17B, and FT10F were grown in CMM-TPGY containing a solid phase of 75ml and a 50ml liquid phase. TPGY broth and DS medium [composed of Modified Duncan-Strong Medium (HiMedia Laboratories Pvt. Ltd., Mumbai, India] prepared according to the manufacturer’s instructions, volume 75ml) were used as controls. The media were inoculated with 75μl of overnight culture in TPGY.

In part 4 of the study, Beluga sporulation mutant strains, a plasmid control strain, and the WT strain were grown in CMM-TPGY supplemented with 15μgml^−1^ thiamphenicol when appropriate.

### Plasmid Design and Mutant Construction

In part 1 of the study, we constructed sporulation mutants of *C. botulinum* Beluga using a novel CRISPR-Cas9 bookmark approach (Seys et al., 2020). All PCR reactions for the cloning procedures were performed using the KOD Hot-Start high fidelity DNA polymerase (Merck). For DNA purification, the GeneJET PCR Purification Kit (Thermo Fisher Scientific, Waltham, MA) or Monarch DNA Gel Extraction Kit (New England Biolabs, Ipswich, MA) was used. Ligation reactions were performed using T4 DNA ligase (Promega Corporation, Madison, WI). For the restriction digestion of DNA, NEB restriction endonucleases were used (New England Biolabs). Plasmid extractions were carried out using the GeneJET Plasmid Miniprep Kit (Thermo Fisher Scientific). Colony PCR screening was performed using the Phusion high-fidelity DNA polymerase (Thermo Fisher Scientific). The DNA concentration was measured using the NanoDrop 1,000 Spectrophotometer (Thermo Fisher Scientific). For DNA size reference in gel electrophoresis, 1kb Plus DNA Ladder was used (New England Biolabs).

All the primers (Metabion International AG) used are listed in Supplementary Table S2. To construct the pMTL431511-Beluga Δ*spo0A*::bm plasmid for generating a Δ*spo0A*::bm genome alteration, we followed a two-step cloning procedure. Firstly, we constructed CRISPR-Cas9 plasmid pMTL431511-Beluga Δ*spo0A*, which served as a template for the final pMTL431511-Beluga Δ*spo0A*::bm plasmid, and carried a copy of a gene encoding truncated but functional Cas9 (Ingle et al., 2019). For the construction of pMTL431511-Beluga Δ*spo0A*, we generated a knockout (KO) cassette consisting of fused, 1,000-bp long regions flanking *spo0A* in the genome, hereafter called the left and right homology arms (LHA and RHA, respectively). The KO cassette was designed to remove the genomic *spo0A* codons 3–270, inclusively, generating an in-frame deletion of *spo0A*. Fragments encoding LHA and RHA were amplified from the genomic DNA of *C. botulinum* Beluga WT strain using two primer pairs (i) F _LHA_*spo0A*-AsiSI with R_LHA_*spo0A* and (ii) F_RHA_*spo0A* with R_RHA_*spo0A*-AscI. Obtained PCR products were purified and fused in splicing by overhang extension-polymerase chain reaction (SOE-PCR) using a pair of primers F_LHA_*spo0A*-AsiSI and R_RHA_*spo0A*-AscI. The resulting DNA fragment contained a complete 2,000-bp *spo0A* KO cassette, flanked by AsiSI and AscI restriction sites. The 20-nt long DNA sequence encoding sgRNA which redirects Cas9 toward *spo0A*, was designed using the CRISPR-Cas9 guide design tool available online in the Benchling platform^1^ with default settings. The pMTL431511-compatible DNA fragment containing template for sgRNA was generated in PCR reaction using the universal 95-nt primer R_sgRNA-AsiSI and customized 107-nt primer F_*spo0A*_sgRNA-SalI which harbored a previously designed template for the sgRNA. The utilized oligonucleotides carried 30-bp overhangs, which enabled their mutual hybridization and thus further nucleotide incorporation to the remaining ssDNA sequence in a PCR reaction. The resulting 172-bp product encoded *spo0A*-specific sgRNA sequence (5'-CATGCTATAGAAGTAGCGTG-3') fused to Cas9-recognized RNA scaffold flanked by restriction sites SalI and AsiSI compatible with the pMTL431511 modular shuttle vector. pMTL431511 linearized with AscI and SalI was ligated with the digested KO cassette and sgRNA-containing fragment in a ratio of 1:3:3, respectively. Chemically competent NEB 5-alpha *E. coli* cells were transformed with the ligation mixture, plated on selective solid LB medium, and incubated overnight at 37°C. Resulting antibiotic-resistant colonies were screened in colony PCR with the primer pair F_P*thl*_scr and 83XXX-LR, which flank the insert-containing plasmid region. Positive colonies were incubated overnight in 5ml of selective LB broth at 37°C with shaking. The plasmid pMTL431511-Beluga Δ*spo0A* was extracted from *E. coli* cultures and sequenced using primer pair F_*spo0A*_seq and R_*spo0A*_seq in Sanger sequencing to confirm the correct insert sequence.

Vector pMTL431511-Beluga Δ*spo0A*::bm was constructed by inserting 24-bp bookmark sequence into the LHA and RHA junction localized on pMTL431511-Beluga Δ*spo0A*. The bookmark sequence was designed to contain an efficient 20-nt Cas9-targeting sequence, 3-nt Protospacer Adjacent Motif (PAM), and an additional adenine to maintain the gene alteration in-frame. Shuttle vector pMTL431511-Beluga Δ*spo0A* was spliced into two fragments in PCR amplification: the first fragment was generated using the F_*spo0A*_bm and R_*cas* primers, and the second with F_*cas* and R_*spo0A*_bm. Primers F_*spo0A*_bm and R_*spo0A*_bm contained complementary overhangs which encoded the bookmark sequence, and were used to generate the insertion into the LHA and RHA junction. The resulting fragments were fused in a NEBuilder reaction (New England Biolabs) following the manufacturer’s recommendations. Positive *E. coli* clones were selected through colony PCR screening using the primer pair F_bm_scr and 83XXX-LR. The obtained plasmid pMTL431511-Beluga Δ*spo0A*::bm was sequenced with Sanger sequencing to verify the correct sequence of the bookmark insertion.

Of note, the final CRISPR-Cas9 vector pMTL431511-Beluga Δ*spo0A*::bm was generated using a two-step procedure where we utilized a previously constructed pMTL431511-Beluga Δ*spo0A* vector available in our laboratory. If no pre-existing vector is available, we would recommend performing a regular one-step cloning.

For the construction of vector pMTL431511-Beluga::*spo0A*-wm, a 2,804-bp complementation cassette was assembled. The fragments encoding LHA and RHA were amplified from the genomic DNA of *C. botulinum* Beluga WT strain, using two primer pairs: (i) F_LHA_*spo0A*-AsiSI with R_*spo0A*_wm, and (ii) F_*spo0A*_wm with R_RHA_*spo0A*-AscI. The resulting DNA fragments were fused in SOE-PCR yielding a full complementation cassette. A sgRNA-encoding bookmark-targeting fragment (5'-GTACGACACCTCGATCACCA-3') was synthesized as described above, using primers F_bm_sgRNA-SalI and R_sgRNA-AsiSI. Ligation of DNA fragments and *E. coli* transformation were performed as described earlier. Positive colonies were screened with colony PCR using primers F_*spo0A*_seq and R_*spo0A*_seq. The constructed plasmid pMTL431511-Beluga *spo0A*-wm was sequenced to confirm the correct insert sequence.

The conjugation donor *E. coli* CA434 (Purdy et al., 2002) was transformed with an appropriate plasmid and grown overnight in liquid selective LB broth at 37°C with shaking. An aliquot of 1ml of stationary-phase overnight culture was washed with 1ml of anaerobic phosphate-buffered saline (PBS). The *E. coli* cell pellet was re-suspended with 200μl of *C. botulinum* overnight culture in an anaerobic chamber. The cell suspension was spotted on an anaerobic TPGY agar plate and incubated to ensure conjugation for 8–10h at 30°C in the anaerobic chamber. The bacterial growth was scraped with a sterile inoculation loop and re-suspended in 500μl of anaerobic PBS which was subsequently spread onto three selective TPGY-agar plates. The plates were incubated at 30°C for 24h in the chamber. The *C. botulinum* mutant colonies resulting from conjugation of *C. botulinum* WT strain with *E. coli* CA434 pMTL431511-Beluga Δ*spo0A*::bm were screened through colony PCR using primers F_*spo0A*_scr and R_*spo0A*_scr. Positive mutant colonies were plated on non-selective TPGY-agar plates anaerobically for 2days at 30°C. Several resulting random colonies were picked and replica-plated on non-selective and selective TPGY agar plates. The clones growing only on non-selective TPGY plates were stored and confirmed by PCR as plasmid-cured. The plasmid loss frequency for *C. botulinum* Beluga strain is around 50–60% of screened colonies.

Primers F_*spo0A*_scr and R_*spo0A*_seq were used to screen for complemented *C. botulinum* colonies resulting from conjugation of *C. botulinum* Δ*spo0A*::bm with *E. coli* CA434 pMTL431511-Beluga::*spo0A*-wm. The PCR products obtained from colony PCR reactions were separated in an agarose gel (1.5% w/v) to verify the amplicon size. After initial confirmation of the complementation, the PCR products derived from positive clones were purified and 600ng of each was digested with PstI-HF (New England Biolabs) for 1h at 37°C. An aliquot of 25μl of the digests were separated in an agarose gel to reveal the resulting digestion pattern. Sanger sequencing confirmed the successful complementation and correct single-nucleotide modifications. CRISPR-Cas9 plasmid was cured from the positive mutant colonies as described above.

### Spore and Viable Cell Count Assays

In parts 2–4 of the study, spore counts were determined using spore heating assays. A 200-μl aliquot of culture was incubated for 20min at 60°C to eliminate vegetative cells. Heat-treated samples were serially diluted in fresh TPGY and, whenever applicable, supplemented with 15μgml^−1^ thiamphenicol, in 96-well plates and incubated anaerobically for 5days at 30°C. The most probable number technique with three tubes was applied to enumerate heat-stable spores (Blodgett, 2000). Non-heated aliquots were similarly investigated to estimate viable cell counts.

### Phase-Contrast Microscopy

Phase-contrast microscopy was used to monitor sporulation 14days after inoculation in parts 3 and 4 of the study. The cultures were gently mixed and 200-μl culture aliquots were centrifuged for 2min at 5000×*g*, the supernatant was discarded, and cell pellet was re-suspended in 30–50μl of anaerobic PBS. A small amount (2μl) of resulting cell suspension was immobilized on a thin layer of 1.7% agarose (SeaKem LE agarose, Lonza) coated on the surface of a microscopy slide. Cells were visualized in DMi8 Leica inverted fluorescent microscope equipped with HC PL APO 100x/1.40 OIL PH3 objective and Hamamatsu Orca Flash V2 LT camera. Microscopy images were captured using the Metamorph Basic Acquisition for Microscope software and adjusted and analyzed with COREL PaintShop Pro x9 and Fiji ImageJ version 1.51, respectively.

### Statistical Analysis

One-way ANOVA was used to compare the total viable cell and spore counts of each strain in different media at different time points in part 3 of the study. Student’s *t*-test was used to compare total viable cell counts or spore counts of mutant and complemented strains to the WT or respective plasmid control strain in part 4 of the study.

## Results and Discussion

### Construction of *C. botulinum* Group II Mutants Using CRISPR-Cas9 Bookmark Approach

The assignment of function to a particular gene product requires that the phenotypic properties of a mutant culture in which the encoding gene has been deleted are compared to those of the progenitor, WT culture. However, the phenotype of the isolated mutant may be a consequence of the acquisition of ancillary mutations elsewhere in the genome during the mutagenesis procedure, which may be responsible for the changed phenotype. To assign the observed phenotype to the absence of the deleted gene, it is essential that a complementation test is performed in which a functional copy of the deleted gene is introduced back into the mutant cells and the phenotype of the complemented mutant shown to be restored to that of the progenitor, WT cell. As the complementation method itself can also cause unintended changes in phenotype, especially if the introduced gene is on an autonomous plasmid, we chose to experimentally validate a recently proposed concept of bookmark complementation (Seys et al., 2020). In essence, the procedure restores the mutant to the WT configuration by replacing the mutant allele in the chromosome with the WT gene. It relies on prior incorporation of a unique 24-nucleotide (nt) bookmark sequence into the mutant allele that acts as a single guide RNA target for its subsequent Cas9-mediated substitution with the WT allele.

**Figure 1:**
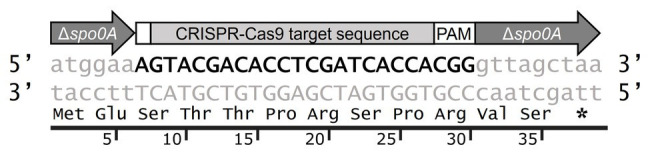
Schematic design of Δ*spo0A*::bm mutation introduced into *Clostridium botulinum* Beluga genome using CRISPR-Cas9 genome modification tool. The 39-bp Δ*spo0A*::bm open reading frame encodes the two first and the two last amino acids of the wild type (WT) *spo0A* (gray arrows), and a bookmark sequence (bold and capitalized sequence). The bookmark sequence consists of an additional adenine (small white segment), a unique CRISPR-Cas9 targeting sequence, and a Protospacer Adjacent Motif (PAM) sequence.

As the first practical demonstration of the bookmark concept (Seys et al., 2020), we chose to target the *spo0A* of our model *C. botulinum* Group II strain Beluga. Accordingly, we used the previously described (Seys et al., 2020) KO system, based on the CRISPR-Cas9 of *Streptococcus pyogenes*, to delete *spo0A* and replace it with a mutant allele comprising a 24-nt bookmark sequence flanked by the first and last two codons of the structural gene. The bookmark (Figure 1) was designed by applying a GC-rich sequence comprised an efficiently cleaved 20-nt CRISPR-Cas9 target (efficiency score calculated using algorithm available on https://www.benchling.com/), 3-bp PAM and an additional base pair maintaining the resulting gene deletion in frame. An nBLAST analysis of the bookmark sequence against the order *Clostridiales* indicated no matches in the genome sequences currently available in public databases, suggesting it could find wide use in these bacteria. By applying this approach, we generated a *C. botulinum* Beluga Δ*spo0A*::bm deletion mutant. Following the transfer of the CRISPR-Cas9 deletion vector, we performed colony PCR screening of 12 randomly picked antibiotic-resistant colonies. Six out of the twelve colonies screened were deletion mutants, and thus the mutant generation success was 50% (Supplementary Figure S1). CRISPR-Cas9 deletion vector was cured from the mutant strain and Sanger sequencing confirmed the expected sequence of the Δ*spo0A*::bm modification (Supplementary Figure S2).

To demonstrate the applicability of the gene bookmarking approach in *C. botulinum*, we restored a functional *spo0A* copy into the original chromosomal locus by redirecting the CRISPR-Cas9 machinery toward the introduced bookmark sequence in the Δ*spo0A*::bm mutant. The functional copy of *spo0A* was not the native gene as this would result in a complemented strain that would be indistinguishable from the progenitor WT cell. Consequently, contamination of a culture with the WT strain would be difficult to rule out (Seys et al., 2020). Instead, the gene used for complementation should contain a “watermark” sequence that would distinguish the complemented clones from the WT (Seys et al., 2020). Importantly, the nucleotide changes made should not cause a change to the encoded protein. The applied approach is to make changes that either create, or remove, a restriction enzyme recognition sequence. This allows the authenticity of the clone to be rapidly established through restriction digestion of an amplified PCR fragment encompassing the region in question (Ng et al., 2013). Here we chose to introduce a watermark sequence, comprising silent changes to five separate codons (267A>T, 270A>T, 273T>A, 285T>A, 288A>T) in the *spo0A* gene (Supplementary Figure S5). When designing the watermark sequence, we considered the codon usage of *C. botulinum* species by replacing the WT codons with similarly used synonymous ones. We retrieved the codon usage table for *C. botulinum* from online codon usage database (Nakamura et al., 2000). For the watermark sequence we used codons with similar usage to the ones originally found in the WT strain applying the following changes: codon GCA to GCT (frequency 0.47 and 0.44), GTA to GTT (frequency 0.49 and 0.42), GGT to GGA (frequency 0.34 and 0.5), ATT to ATA (frequency 0.40–0.54), and ACA to ACT (frequency 0.45–0.46).

The watermark lacks the PstI-recognized nucleotide sequence found within the original *spo0A* sequence. Altogether, the introduction of a watermark aims at leaving a genomic imprint in the restored gene copy and facilitates the distinction of complemented from the wild type strain by revealing different digestion patterns of the colony PCR generated DNA fragments. Following transfer of the requisite CRISPR-Cas9 complementation vector into the Δ*spo0A*::bm mutant, 12 antibiotic-resistant transconjugants were selected and all (100%) were shown by colony PCR (Supplementary Figure S3) and PstI digestion (Supplementary Figure S4) to be complemented watermark-harboring strains. Sanger sequencing of a random selection of clones confirmed that the expected region of the DNA had been inserted (Supplementary Figure S5). The watermark sequence applied in this study removes an already existing restriction enzyme site. However, this approach might not be readily applicable due to the limited restriction enzyme availability. In such case, the watermark-specific screening could be performed through PCR. One of the primers applied for the amplification should directly anneal to the watermarked genomic sequence with altered nucleotides at the 3' end, which would increase the specificity of the primer. That way, the watermark-specific primer, together with another regular primer annealing nearby, would yield a PCR product only in the case of successful watermark insertion. However, we would recommend validating the watermark-specific primer before the actual mutant screening to ensure the specificity of primer and expected amplification result.

The successful creation of *C. botulinum* Δ*spo0A*::bm and Δ*spo0A*::bm::*spo0A*-wm strains served to illustrate how the utility of the bookmark approach transcends simple complementation and can be employed to deliver derivatized genes to the genome. In this case, the modified gene contained five silent nucleotide changes, but could equally be applied to incorporate alterations in encoded amino acids for the purposes of structural or mechanistic studies. Successful complementation of *spo0A* harboring the pre-designed nucleotide changes provides a foundation for utilizing the bookmark approach to introduce other genomic alterations within a copy of a restored gene, i.e., modifications of putative protein binding boxes, single-amino-acid substitutions or deletions, or construction of reporter gene fusions.

### Selection of Efficient Sporulation Media for *C. botulinum* Group II

An integral part of spore research is the availability of a laboratory medium that ensures sufficient and reproducible spore yields and which enables downstream processing. Roberts (1967) identified three different media likely support clostridial sporulation: (i) cooked meat medium; (ii) blood agar; and (iii) TPAY-GT composed of tryptone, peptone, ammonium sulfate, yeast extract, glucose, and sodium thioglycolate. No single medium yielded satisfactory results for all tested *C. botulinum* strains (Roberts, 1967). TPGY, a variation of TPAY-GT, eventually became the routine growth medium for *C. botulinum*, and also provided a functional sporulation medium for Group I strains (Eklund et al., 1969). However, while effectively supporting growth, TPGY is not an ideal sporulation medium for Group II strains (Mascher et al., 2017).

Different variations of cooked meat medium have been used to sporulate *C. botulinum* (Perkins, 1965; Roberts, 1967; Solomon and Kautter, 1979), despite difficulties in downstream processes caused by small meat particles floating in the medium (Perkins, 1965; Roberts, 1967). Peck (1992) used a biphasic sporulation medium consisting of a solid phase of CMM supplemented with agar and glucose, and a liquid phase of sterile water, to collect Group II spores for purification and heat inactivation assays (Peck et al., 1992). Other biphasic media with different solid phases and a liquid phase of sterile water have also been shown to support sporulation of Group II strains (Perkins, 1965; Bruch et al., 1968; Peck et al., 1992). The mechanism of a biphasic medium supporting efficient sporulation remains unclear, but it is possible that attachment to a solid surface induces sporulation (Vlamakis et al., 2008). Liquid phases of nutrient-rich broths likely support growth better than a water phase, and thus we expected nutrient-rich liquid phases primarily to yield a larger number of sporulation-prone cells than water (Peck et al., 1992; Braconnier et al., 2001; Brunt et al., 2018).

Our goal was to find a *C. botulinum* Group II sporulation medium that would be straightforward to prepare, support efficient growth and sporulation, and enable easy and particle-free sampling. Therefore, we first screened the growth and sporulation of *C. botulinum* Group II strain Beluga in five different biphasic media: TPGY-H_2_O, EYA-H_2_O, RCM-H_2_O, CMM-H_2_O, and CMM-TPGY. Agar-TPGY and agar-H_2_O were used as controls. All media except for agar-H_2_O supported the growth and sporulation of Beluga, with concentrations of approximately 10^7^–10^8^ cells or spores/ml being reached in all of them (Supplementary Figure S6). However, the solid phase of TPGY-H_2_O, EYA-H_2_O, and RCM-H_2_O was prone to crumbling, causing pipette tips to become clogged by small agar pieces during sampling and considerably interfered with downstream processes, preventing the collection of spores by centrifugation. CMM-H_2_O, CMM-TPGY, and agar-TPGY all enabled effortless sampling, and CMM-TPGY resulted in better growth and subsequently higher spore concentration than the other two media (approximately 1 log, Supplementary Figure S6). This indicates that nutrient-rich solid and liquid phases support growth better than poorer media and, as a result, enable higher spore yields, even if there are no differences in the sporulation rate (maximum spore count divided by the maximum total cell count). Fulfilling our criteria better than the other media, CMM-TPGY was selected for further validation.

**Figure 2:**
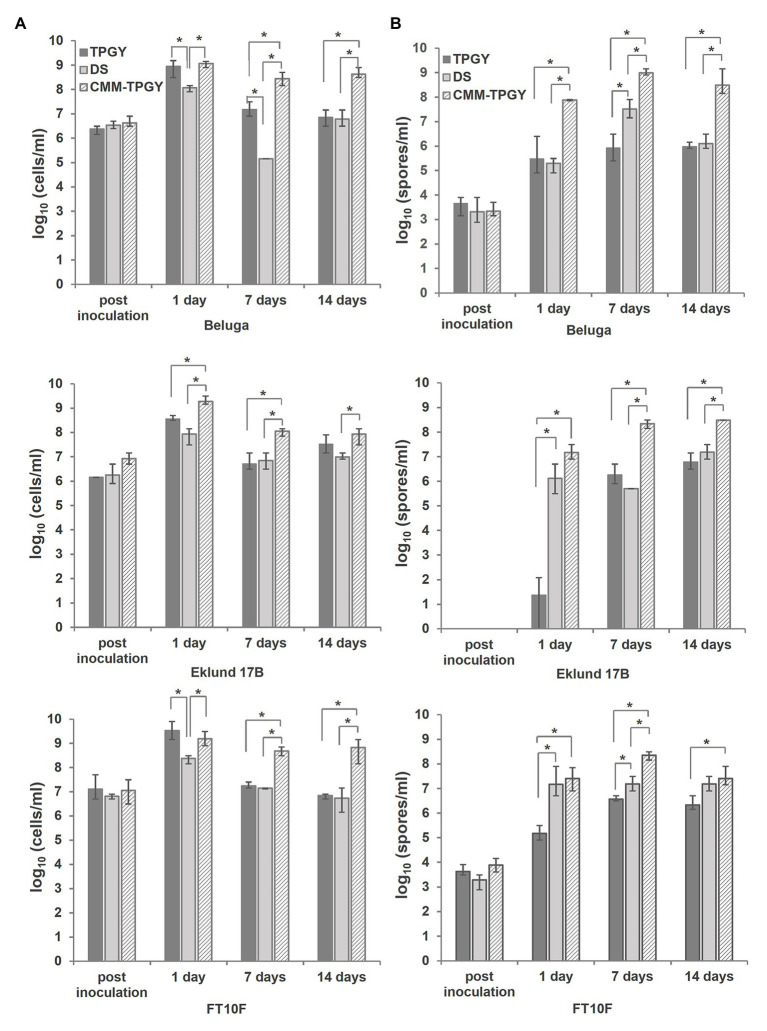
Comparison of viable cell and spore counts produced by three different *C. botulinum* Group II strains (Beluga, Eklund 17B and FT10F) grown in three challenged media: standard tryptone-peptone-glucose-yeast extract (TPGY) broth, *Clostridium perfringens* sporulation medium Duncan-Strong (DS), and the newly described biphasic cooked meat medium-TPGY (CMM-TPGY) broth. Total viable cell and spore enumeration **(A)** and spore heating assays **(B)** were performed directly after inoculating the fresh media, and subsequently at day 1, day 7, and day 14 post inoculation. Biphasic CMM-TPGY medium promoted most efficiently bacterial growth **(A)** and sporulation **(B)** of all three tested *C. botulinum* Group II strains. The experiment was performed in three parallel biological replicates. The error bars represent the maximum and minimum values of three replicates. *Statistically significant (*p*<0.05) difference in cell or spore concentration between different media.

To evaluate the suitability of CMM-TPGY as a sporulation medium for different Group II *C. botulinum* strains, we used it to culture Beluga (toxin type E1), Eklund 17B (toxin type B4) and FT10F (toxin type F6) for 14days at 30°C under anaerobic conditions. The standard TPGY broth (Solomon and Lilly, 2001) and DS used to sporulate *C. perfringens* (Duncan and Strong, 1968) were used as controls. We collected samples for viable cell and spore enumeration immediately after inoculation, day 1, day 7, and day 14. At the end of the growth period, we performed phase-contrast microscopy of the cultures. All three strains reached their maximum total viable cell counts 1day after inoculation in all different media. The growth of Beluga and FT10F was best supported by TPGY broth and CMM-TPGY, with significantly lower (*p*<0.05) total cell count detected in DS broth (Figure 2A). The growth of Eklund 17B was best supported by CMM-TPGY, with significantly lower total cell counts reached in TPGY and DS broths (Figure 2A). Although TPGY broth supported vegetative growth (Figure 2A, day 1), especially in case of strains Beluga and FT10F, sporulation in TPGY was generally poor. All three Group II strains reached significantly higher (*p*<0.05) spore concentrations in the biphasic CMM-TPGY medium than in TPGY broth alone on days 1, 7, and 14 (Figure 2B). The sporulation rates were similar in CMM-TPGY and DS broths for all strains, but due to higher total cell counts, the maximum spore concentrations reached in CMM-TPGY were significantly higher (*p*<0.05) than those in DS. The results indicate that abundant growth leading to a concentrated culture (Tyrrell et al., 1958) in combination with potential factors supporting sporulation, such as attachment (Vlamakis et al., 2008), biofilm formation (Branda et al., 2001), or sporulation-activating cell-to-cell communication due to cell crowding (Cooksley et al., 2010; Li et al., 2011) are prerequisites for a high spore yield.

**Figure 3:**
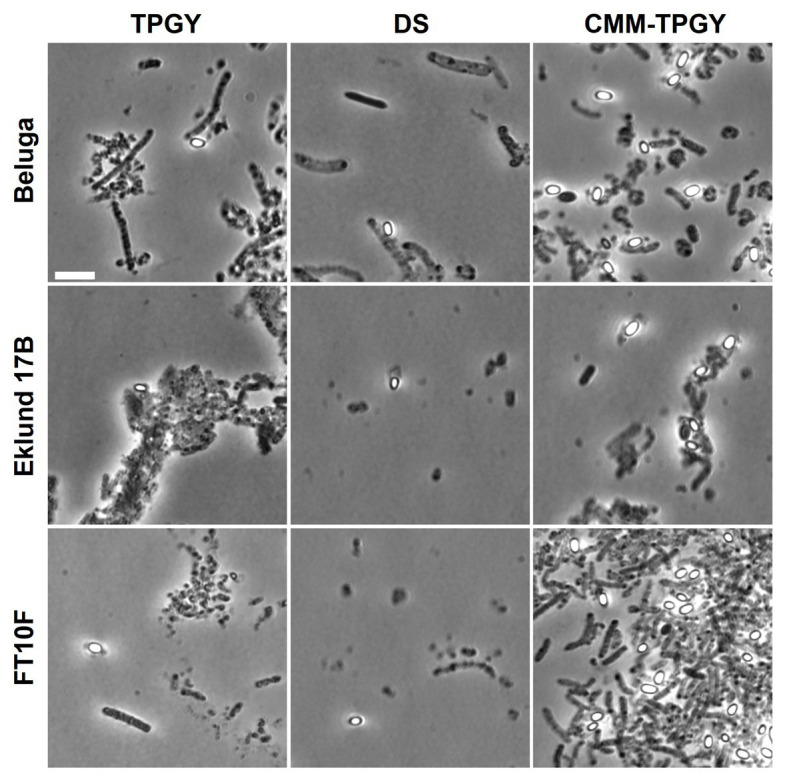
Phase-contrast microscopy of *C. botulinum* Group II strains Beluga, Eklund 17B and FT10F after 14days of growth in TPGY broth, DS broth, and in biphasic CMM-TPGY medium. From the three tested media, the biphasic CMM-TPGY provides the most favorable sporulation conditions for all the tested Group II strains. Scale bar 5μm.

Phase-contrast microscopy of Beluga, Eklund 17B and FT10F grown in CMM-TPGY, TPGY, and DS (Figure 3) supported the results of spore heating assays. Cultures grown in CMM-TPGY showed an abundance of phase-bright mature spores, as opposed to vast amounts of vegetative cell debris and only few spores observed in TPGY, and little cell debris and few spores observed in DS.

### Characterization of Constructed CRISPR-Cas9 Mutants in CMM-TPGY Medium

To characterize the phenotype of the *spo0A* mutants, we performed a comprehensive sporulation test utilizing the biphasic CMM-TPGY medium for cultivating *C. botulinum* Group II Beluga strains Δ*spo0A*::bm and Δ*spo0A*::bm::*spo0A*-wm. Spo0A functions as a master regulator of sporulation in all spore-forming bacteria (Hoch, 1993; Al-Hinai et al., 2015), including *C. botulinum* Group II (Mascher et al., 2017). We therefore expected the *spo0A* deletion to prevent the cells from entering sporulation. Since spore formation can be readily traced in the laboratory, an asporogenous phenotype would serve as a reliable control for successful gene knock-out and for *in cis* or *in trans* complementation of the mutations. To compare the efficiency of a chromosomal gene complementation to the conventional plasmid complementation approach, we constructed a *C. botulinum* Beluga Δ*spo0A*::bm-pMTL82151::*spo0A* strain harboring shuttle vector with a WT copy of *spo0A* under the control of its native promoter (Mascher et al., 2017).

To provide suitable control strains capable of growing in a selective medium, along with the Δ*spo0A*::bm-pMTL82151::*spo0A* strain, we also characterized the WT-pMTL82151 and Δ*spo0A*::bm-pMTL82151 control strains, both carrying just the pMTL82151 shuttle vector. All the strains were cultivated for 2weeks in CMM-TPGY medium in three biological replicates. The plasmid-carrying cultures were supplemented with 15μgml^−1^ thiamphenicol every 48h to maintain sufficient concentration of antibiotic during the extended incubation.

**Figure 4:**
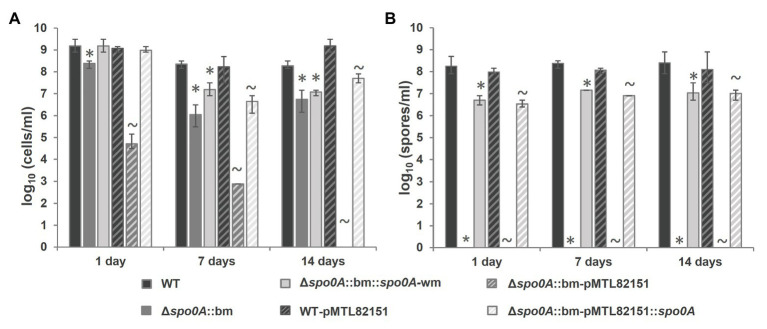
Phenotype characterization in CMM-TPGY sporulation medium of the constructed Δ*spo0A*::bm *C. botulinum* Beluga strain compared to the WT parental and complemented strains, where a copy of functional *spo0A*-coding sequence was inserted back to the original chromosomal locus (Δs*po0A*::bm::*spo0A*-wm) or expressed from a plasmid (Δ*spo0A*::bm-pMTL82151::*spo0A*). **(A)** Viable cell and **(B)** spore quantifications were performed at day 1, day 7, and day 14 post inoculation. The Δs*po0A*::bm mutant strain produced significantly smaller total viable cell counts than the WT strain **(A)** due to the inability to form heat-resistant spores **(B)**. Both complementation strategies allowed comparable restoration of asporogenous phenotype for Δs*po0A*::bm. All the data were obtained from three biological replicates. *Cell or spore concentration of a mutant or complemented strain was significantly (*p*<0.05) lower than that of WT. ~Cell or spore concentration was significantly (*p*<0.05) lower than that of the respective plasmid control.

**Figure 5:**
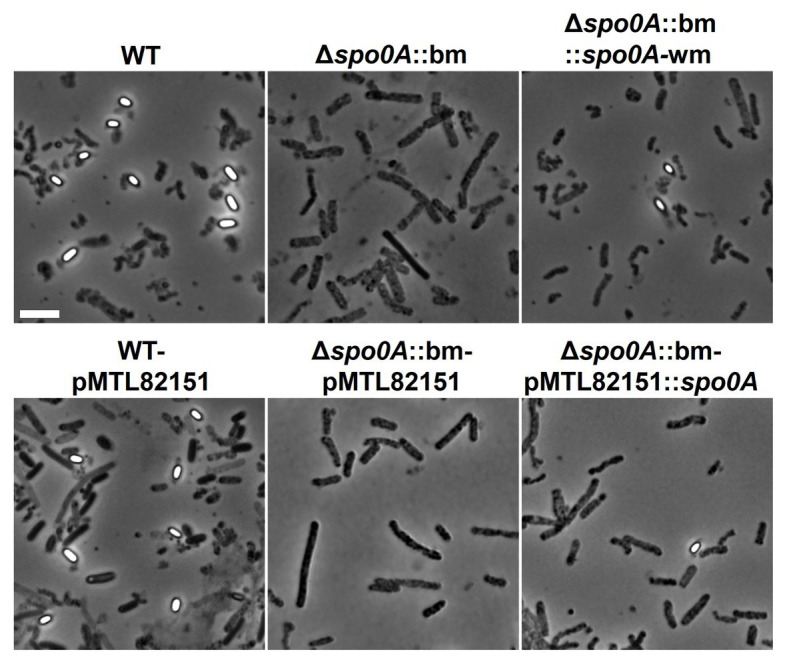
Phase-contrast microscopy images at 14days post inoculation demonstrating the morphological differences between WT, Δs*po0A*::bm, and complemented mutant strains. Phase-bright endospores were detectable only in samples containing WT or complemented strains. The Δs*po0A*::bm mutant did not display any spores or sporulating cells, suggesting that endospore formation ceased at the early stage. Scale bar 5μm.

As expected, the mutant Δ*spo0A*::bm was unable to enter sporulation, which was verified by the absence of heat-resistant spores (Figure 4B) and the visual absence of sporulating cells or free spores when subjected to microscopic examination (Figure 5). The maximum average cell count reached in the mutant cultures, 2.54×10^8^ cells/ml, was slightly but significantly (*p*=0.039) lower than that of the WT strain (1.78×10^9^ cells/ml). As the applied total cell count assay enumerates the sum of vegetative cells and spores, a lower concentration of cells in the Δ*spo0A*::bm culture is likely due to lack of spores. The sporulating phenotype was successfully restored by returning a watermark-harboring *spo0A* copy in the original *spo0A* locus applying the CRISPR-Cas9 bookmark-targeting approach. The complemented Δ*spo0A*::bm::*spo0A*-wm mutant showed no significant differences to WT in the maximum cell concentrations, but the maximum spore counts were approximately 1 log lower in the complemented strains when compared to WT (*p*<0.001; Figure 4A). The difference in sporulation rate between WT and complemented Δ*spo0A*::bm::*spo0A*-wm strain could arise from the limitations of the applied MPN enumeration method that is a statistical approach giving only an estimate number of cells in bacterial suspension, and it is known to be less accurate in case of dense bacterial populations (Chandrapati and Williams, 2014). Another explanation for the observed difference in spore number would be an effect of secondary SNPs in the genome, presumably caused by stress during the mutational process. Nevertheless, the spores of the complemented strains are produced in relatively high amount; they are heat-resistant and show regular morphology indicating that the entire sporulation pathway is restored and functional.

The plasmid-borne complementation of the *spo0A* deletion was equally efficient (reaching approximately 10^7^ spores/ml) as the *spo0A*-wm insertion into the chromosomal *spo0A* locus, indicating that the introduction of a bookmark (i) does not interfere with the phenotype, (ii) is unlikely to cause polar effects, and (iii) can be safely used for routine deletion-tagging. No differences in growth or sporulation were detected between WT and its plasmid control strain WT-pMTL82151 (Figures 4A,B). However, the second control strain Δ*spo0A*::bm-pMTL82151 showed significantly lower total cell numbers than the respective mutant Δ*spo0A*::bm (*p*<0.001) and no growth at all on day 14. A possible explanation behind this observation could be a gradual plasmid loss rendering the strain increasingly sensitive to thiamphenicol. In case of the sporulation-deficient strain incubated during extended periods, the control plasmid cannot be preserved inside the spores, therefore, the bacteria lose the ability to grow in antibiotic-supplemented broth.

## Conclusion

The present study represents the first use of CRISPR-Cas9 in the generation of in-frame deletions in *C. botulinum* Group II. The system used relies on a truncated Cas9 nickase variant previously only exemplified in *Clostridium difficile* (Ingle et al., 2019). Using the *spo0A* gene as the target, we also provided the first practical demonstration of CRISPR-Cas9-mediated bookmark complementation technology (Seys et al., 2020), in which the deleted chromosomally located *spo0A* gene was restored to a functional copy *in situ*. This substitution was made possible by the prior incorporation into the mutant allele of a 24-nt bookmark sequence that should be widely applicable in other clostridial species. The functional copy of *spo0A* used for complementation was “watermarked” with five silent nucleotide changes that removed a WT PstI restriction site but still enabled production of active Spo0A. This was to allow the complemented strain to be easily distinguished from possible contamination with the WT, but also to demonstrate the potential of the bookmark technology in functional studies in which a chromosomal gene is replaced, in a two-step process, with a derivatized copy. In future studies such genes could include, for instance, variants with specific amino acid changes, homologs from other bacterial strains or species, and fusion proteins. Our analysis of the sporulation mutant and the generated complemented strain was made possible by the formulation of a biphasic CMM-TPGY medium, which supported the growth and efficient sporulation of three different *C. botulinum* Group II strains and enabled easy and particle-free sampling for microscopy and other downstream applications. Altogether, the findings described in this study constitute a solid base for future spore and sporulation studies of *C. botulinum* Group II strains.

## Data Availability Statement

The original contributions presented in the study are included in the article/Supplementary Material, further inquiries can be directed to the corresponding author.

## Author Contributions

Conceptualization: MBN, AM, GM, and ML. Formal analysis: MBN and AM. Investigation: MBN, AM, GM, and VH. Methodology: MBN, AM, GM, DG, and NPM. Writing the original draft—MBN, AM, and ML. Reviewing and editing the original draft: all authors. All the authors have read and agreed on the published version of the manuscript.

### Conflict of Interest

The authors declare that the research was conducted in the absence of any commercial or financial relationships that could be construed as a potential conflict of interest.

## Footnotes

^1^https://www.benchling.com/
