# Supplementary material for: CRISPR-Cas9-Based Toolkit for Clostridium botulinum Group II Spore and Sporulation Research
Source: Front Microbiol. 2021 Jan 27;12:617269. doi: 10.3389/fmicb.2021.617269 (PMC7873358; doi:10.3389/fmicb.2021.617269)
Supplement: Supplementary file 1 [file Data_Sheet_1.docx]

**Supplementary Table 1.** Strains and plasmids used.

| **Strains/plasmids** | **Description** | **Source** |
| --- | --- | --- |
| ***Clostridium botulinum*** |  |  |
| FT10F | Wild type strain isolated from herring | ATCC^a^ |
| Eklund 17B | Wild type strain isolated from marine sediment | IFR^b^ |
| Beluga | Wild type strain isolated from fermented Beluga whale flippers | IFR |
| Beluga Δ*spo0A*::bm | In-frame deletion mutant of *spo0A* with insertion of bookmark sequence. Deletion includes amino acids 3-270. | This study |
| Beluga Δ*spo0A*::bm::*spo0A*-wm | In-frame deletion mutant of *spo0A* with chromosomal complementation of *spo0A* gene harboring five customized nucleotide changes (‘watermarks’) | This study |
| Beluga-pMTL82151 | Wild type strain with empty complementation plasmid | This study |
| Beluga Δ*spo0A*::bm-pMTL82151 | Mutant strain Δ*spo0A*::bm with empty complementation plasmid | This study |
| Beluga Δ*spo0A*::bm- pMTL82151::*spo0A* | Mutant strain Δ*spo0A*::bm with *spo0A* complementation plasmid | This study |
| ***Escherichia coli*** |  |  |
| CA434 | Conjugation donor | Purdy et al. |
| NEB 5-alpha | Cloning strain | New England BioLabs |
| **Plasmids** |  |  |
| pMTL431511 | Empty CRISPR-Cas9 vector | Ingle et al. |
| pMTL431511-Beluga Δ*spo0A* | CRISPR-Cas9 vector for constructing *spo0A* in-frame deletion | This study |
| pMTL431511-Beluga Δ*spo0A*::bm | CRISPR-Cas9 vector for constructing *spo0A* in-frame deletion labelled with the bookmark sequence | This study |
| pMTL431511-Beluga::*spo0A*-wm | CRISPR-Cas9 vector targeting the bookmark sequence and replacing it with watermark-including *spo0A* sequence | This study |
| pMTL82151 | Empty complementation plasmid | Heap et al. |
| pMTL82151::*spo0A* | Complementation plasmid encoding Beluga *spo0A* wild type gene together with 422-bp of genome present upstream and 143-bp downstream *spo0A* | Mascher et al. |

^a^American Type Culture Collection

^b^Culture Collection of the Institute of Food Research, Norwich, UK

Supplementary Table 2. Primers used. The underlined sequences indicate restriction enzyme cleavage sites.

| **Primer name** | **Sequence (5´→3´)** | **Information** |
| --- | --- | --- |
| F _LHA_*spo0A*-AsiSI | GTATACGCGATCGCTAGGTACAATGACTTTTATAGACGGTG | Construction of the left homology arm for pMTL431511-Beluga Δ*spo0A* |
| R_LHA_*spo0A* | ATTAGCTAACTTCCATGCTTTTTCCTCTCCTTC | Construction of the left homology arm for pMTL431511-Beluga Δ*spo0A* |
| F_RHA_*spo0A* | AAGCATGGAAGTTAGCTAATCGTAGTGATATCAATG | Construction of the right homology arm for pMTL431511-Beluga Δ*spo0A* |
| R_RHA_*spo0A*-AscI | ATATCTGGCGCGCCTTACGTAATCCATGTGGAGTTATATTC | Construction of the right homology arm for pMTL431511-Beluga Δ*spo0A* |
| R_sgRNA-AsiSI | CGCGCGCGGCGATCGCATAAAAATAAGAAGCCTGCAAATGCAGGCTTCTTATTTTTATAAAAAAAGCACCGACTCGGTGCCACTTTTTCAAGTTG | Construction of a fragment containing sgRNA template |
| F_*spo0A*_sgRNA-SalI | TTTTCGTCGACCATGCTATAGAAGTAGCGTGGTTTTAGAGCTAGAAATAGCAAGTTAAAATAAGGCTAGTCCGTTATCAACTTGAAAAAGTGGCACCGAGTCGGTGC | Construction of a fragment containing *spo0A*-specific sgRNA template |
| F_*spo0A*_bm | AGTACGACACCTCGATCACCACGGGTTAGCTAATCGTAGTGATATCAATGGTTTAGAG | Insertion of the bookmark sequence into pMTL431511-Beluga Δ*spo0A* plasmid |
| R_*spo0A*_bm | CCGTGGTGATCGAGGTGTCGTACTTTCCATGCTTTTTCCTCTCCTTCATAAAC | Insertion of the bookmark sequence into pMTL431511-Beluga Δ*spo0A* plasmid |
| F_*cas* | CACAAACAATTGTTTTTGTTCGTTATCTTC | Splicing of pMTL431511-Beluga Δ*spo0A* plasmid |
| R_*cas* | GAAGATAACGAACAAAAACAATTGTTTGTG | Splicing of pMTL431511-Beluga Δ*spo0A* plasmid |
| F_*spo0A*_wm | CTGCTGTTGGACAAGATAAGATAACTCAACAAGCAATAACATTAGGTGC | Construction of a complementation plasmid pMTL431511-Beluga::*spo0A-*wm |
| R_*spo0A*_wm | GTTGAGTTATCTTATCTTGTCCAACAGCAGATAATATTATAATTTTAGGCATTTTTTC | Construction of a complementation plasmid pMTL431511-Beluga::*spo0A-*wm |
| F_bm_sgRNA-SalI | TTTTCGTCGACGTACGACACCTCGATCACCAGTTTTAGAGCTAGAAATAGCAAGTTAAAATAAGGCTAGTCCGTTATCAACTTGAAAAAGTGGCACCGAGTCGGTGC | Construction of a fragment containing bookmark-specific sgRNA template |
| F_P*thl*_scr | GATACGGGGTAACAGATAAACC | Sequencing and screening of pMTL431511 plasmids series |
| 83XXX-LR | ACGGCTTGATGTGTTGGTAG | Sequencing and screening of pMTL431511 and pMTL83XXX plasmids series |
| F_*spo0A*_seq | GTAGTTTATGATGTTATAGGGATTGTG | Sequencing of *spo0A* locus and screening |
| R_*spo0A*_seq | GAAATTCTAGGCAATAAAAAATACACCTCC | Sequencing of *spo0A* locus and screening |
| F_*spo0A*_scr | GGGTGGAAATAGTGTTGGTG | Screening of *spo0A* deletion |
| R_*spo0A*_scr | CCCTAGAATCTTAGATAAAATCATTAATG | Screening of *spo0A* deletion |


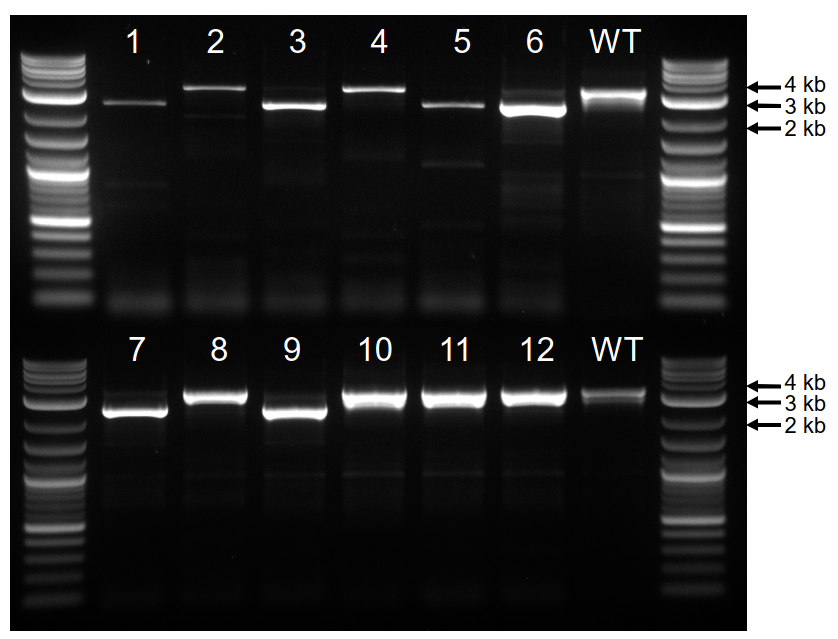


Supplementary Figure 1. Agarose gel electrophoresis of PCR products from twelve antibiotic-resistant *Clostridium botulinum* Beluga wild type (WT) strain colonies transformed with pMTL431511-Beluga Δ*spo0A*::bm plasmid. The targeted *spo0A* locus was amplified with primer pair F_*spo0A*_scr and R_*spo0A*_scr and the resulting products were separated in agarose gel (1.5% w/v). The expected size of the amplicon harboring the designed Δ*spo0A*::bm deletion is 2.4 kb and the wild type amplicon size is 3.1 kb. Six out of twelve screened colonies were confirmed to be desired mutants (lanes 1, 3, 5, 6, 7, 9). Hence, the Δ*spo0A* deletion generation success is estimated to be 50%.


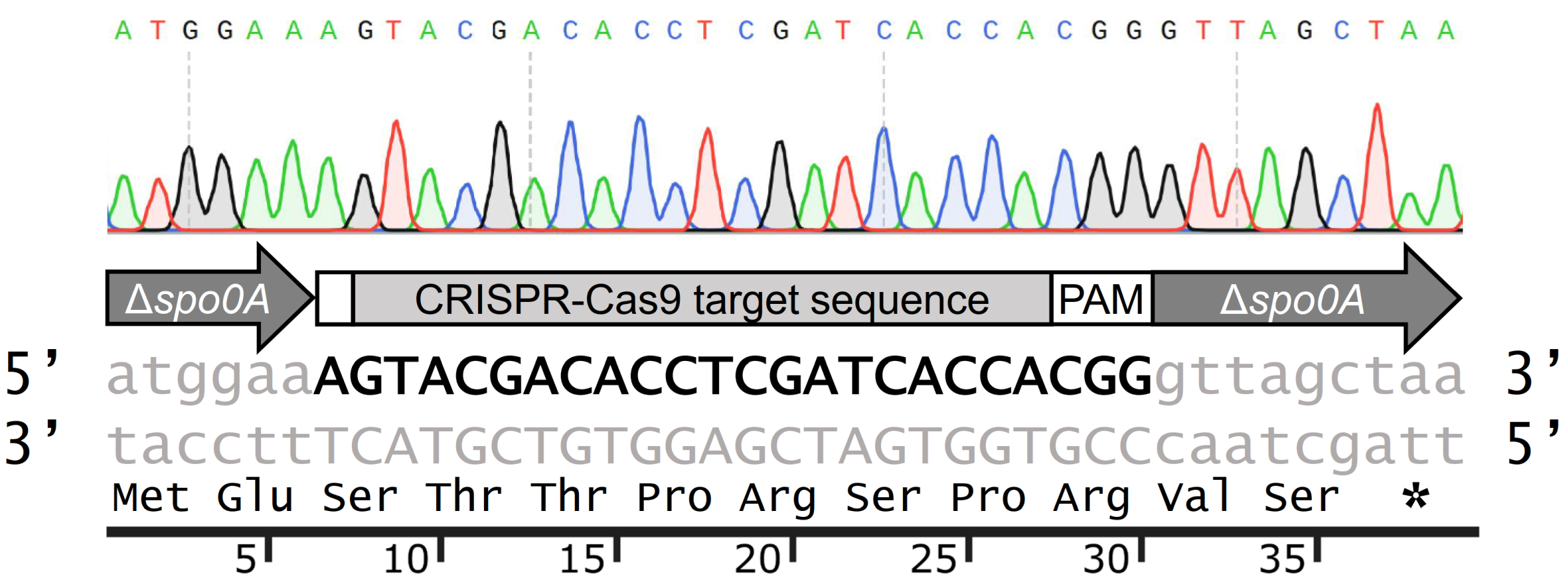


Supplementary Figure 2. Sanger sequencing chromatogram of the PCR-amplified fragment from the genome of constructed *C. botulinum* Beluga Δ*spo0A*::bm aligned with the designed sequence of Δ*spo0A*::bm modification.

**
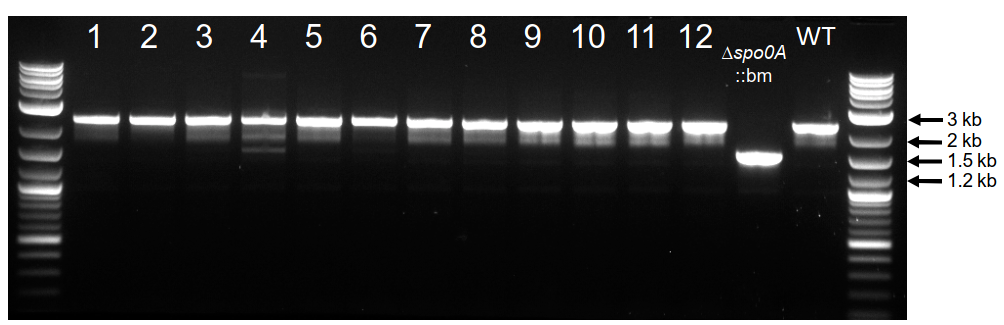
**

**Supplementary Figure 3.** Agarose gel electrophoresis of PCR products from twelve antibiotic-resistant *C. botulinum* Beluga Δ*spo0A*::bm strain colonies transformed with complementation plasmid pMTL431511-Beluga Δ*spo0A*::bm::*spo0A*-wm. The targeted *spo0A* locus was amplified with primer pair F_*spo0A*_scr and R_*spo0A*_seq separated in agarose gel (1.5% w/v). The expected size of amplicon harboring the chromosomal complementation is 2.3 kb and the Δ*spo0A*::bm deletion amplicon is 1.5 kb. All twelve colonies screened were confirmed to be the desired complemented mutants.


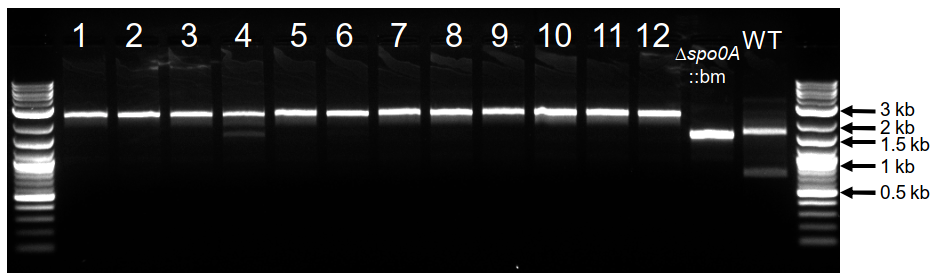


Supplementary Figure 4. Agarose gel electrophoresis of PstI-digested PCR products from twelve antibiotic-resistant clones of *C. botulinum* Beluga Δ*spo0A*::bm::*spo0A*-wm. The targeted *spo0A* locus was amplified using primer pair F_*spo0A_*scr and R_*spo0A*_seq. Equal amount of the resulting purified products were digested with PstI and separated in agarose gel (1.5% w/v). The watermark introduced into the complemented copy of *spo0A-*wm removes PstI restriction site unique for wild type *spo0A*. Therefore, the colonies harboring *spo0A*-wm demonstrate an undigested 2.3 kb PCR product. The wild type PCR product harbors PstI restriction site showed as a cleavage of 2.3 kb PCR product into two fragments 1,600-bp and 700-bp long. All twelve colonies screened were confirmed to be the desired complementation mutants harboring a pre-designed watermark, resulting in 100% success of the complementation procedure.


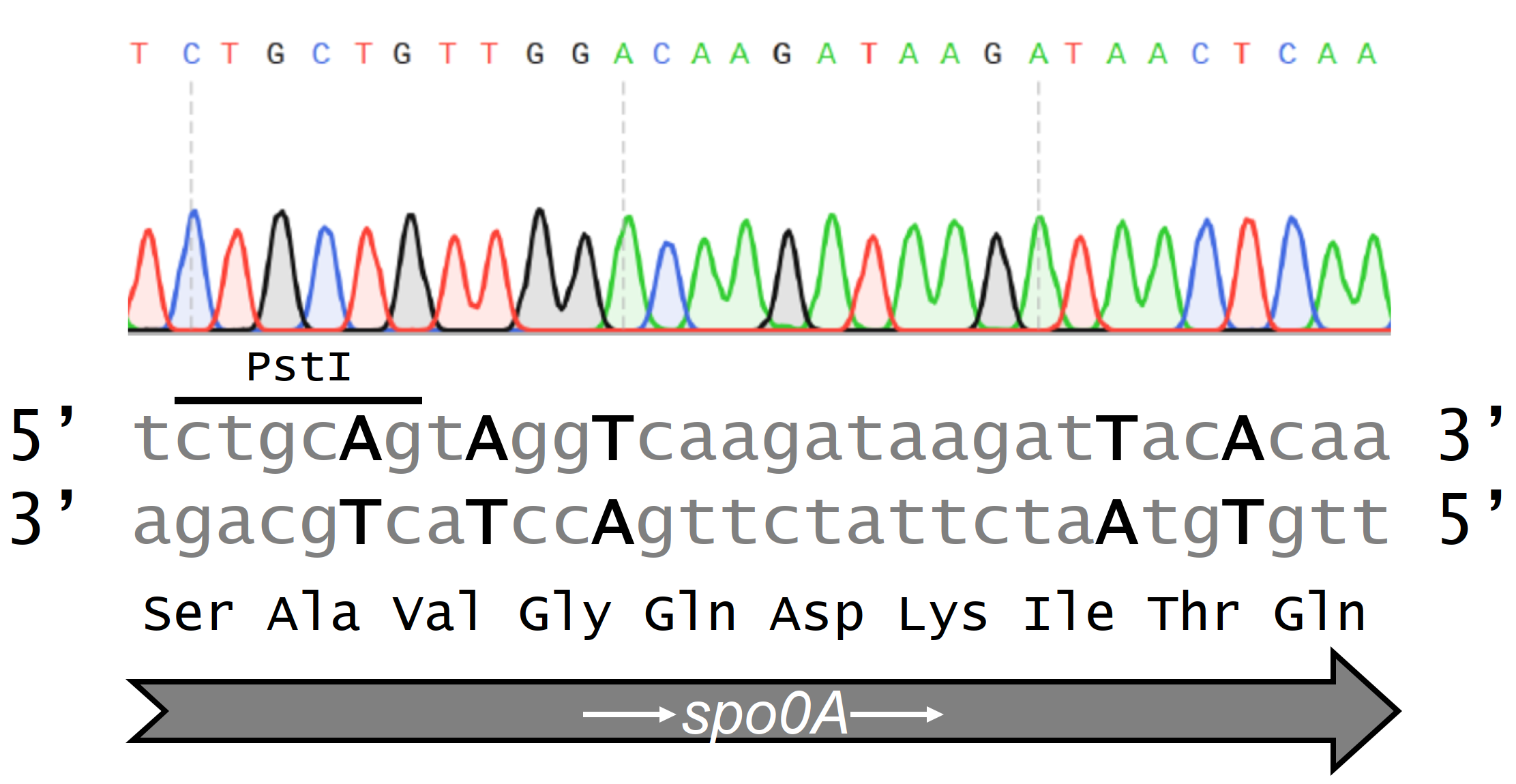


Supplementary Figure 5. Sanger sequencing chromatogram of the PCR-amplified fragment from the genome of complemented *C. botulinum* Beluga Δ*spo0A*::bm::*spo0A*-wm strain. Chromatogram is aligned with the fragment of wild type *spo0A* sequence (including 265-291 -bp of the coding sequence)*.* The altered base pairs are capitalized (267A>T, 270A>T, 273T>A, 285T>A, 288A>T). The black line indicates the PstI restriction site unique for the wild type *spo0A* sequence.


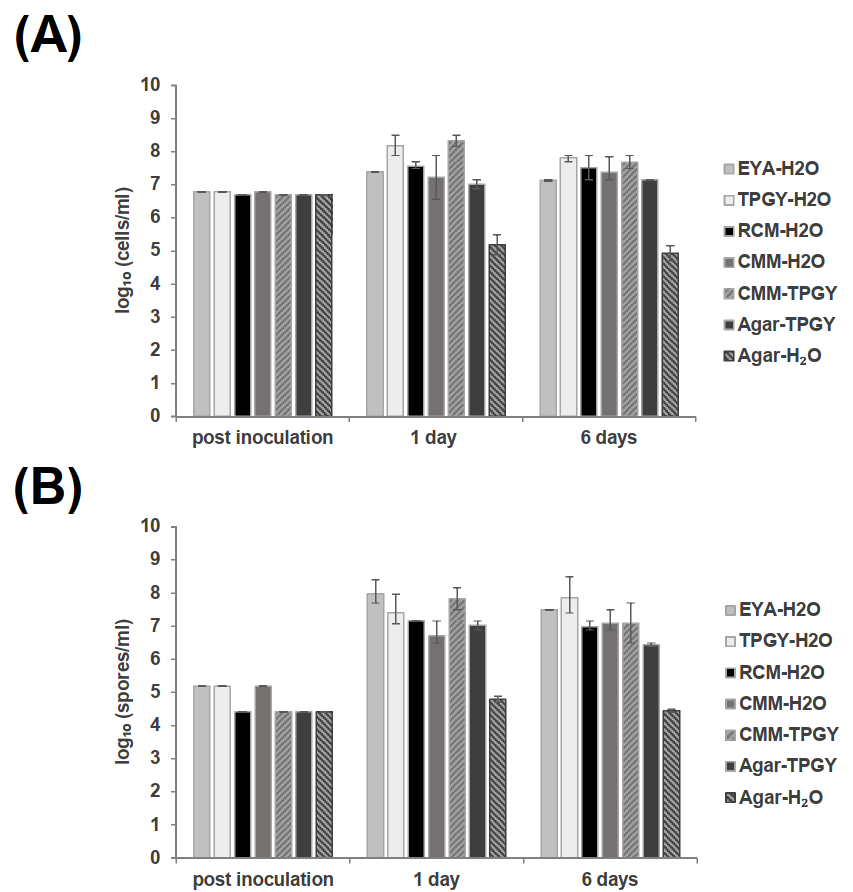


Supplementary Figure 6. Growth and sporulation of *C. botulinum* Group II strain Beluga in different biphasic media: Egg yolk agar (EYA)-H_2_O, tryptone-peptone-glucose-yeast extract (TPGY) agar-H_2_O, Reinforced Clostridial Medium (RCM) agar-H_2_O, cooked meat medium (CMM)-H_2_O, CMM-TPGY, agar-TPGY, and agar-H_2_O. All media had a 150-ml solid phase and a 20-ml liquid phase, the latter inoculated with 5 ml of overnight culture grown in TPGY. Total viable cell enumeration (A) and spore heating assay (B) were performed directly after inoculation and one and six days after inoculation. Error bars represent minimum and maximum values of three replicates.
